# A Scoping Review to Map Health Outcomes in Inducible Laryngeal Obstruction

**DOI:** 10.1111/1460-6984.70169

**Published:** 2025-12-17

**Authors:** S. F. Ludlow, L. J. Holmes, L. Simpson, L. Byrne‐Davis, S. J. Fowler

**Affiliations:** ^1^ Manchester Airways Service Manchester University NHS Foundation Trust Manchester UK; ^2^ Medical Education, School of Medical Sciences, Faculty of Biology, Medicine and Health University of Manchester Manchester UK; ^3^ Manchester Severe Asthma Service Manchester University NHS Foundation Trust Manchester UK; ^4^ The School of Nursing Faculty of Biology, Medicine and Health University of Manchester Manchester UK; ^5^ Patient and Public Involvement (PPI) Manchester University NHS Foundation Trust Manchester UK; ^6^ Manchester Severe Asthma and Airways Service Manchester University NHS Foundation Trust Manchester UK; ^7^ Division of Infection, Immunity and Respiratory Medicine, School of Biological Sciences University of Manchester Manchester UK

**Keywords:** evidence synthesis, inducible laryngeal obstruction, health outcomes, patient‐reported outcome measure

## Abstract

**Background:**

Inducible laryngeal obstruction (ILO) is defined as an inappropriate laryngeal closure causing difficulty in breathing. Outcome measures can be used in ILO to monitor changes in health status over time. A comprehensive review of existing measures is important to understand what the targets of treatment and management are and whether there is a need for development of new tools.

**Aims:**

To systematically evaluate the literature reporting outcomes in individuals with ILO, identifying what is measured, whether there is consistency of measurement to enable synthesis of evidence for treatment and management, and whether measurement considers the areas of body function and structure, activity and contextual factors in line with the World Health Organisation International Classification of Functioning, Disability and Health framework (WHO‐ICF).

**Methods:**

A systematic search was conducted of MEDLINE, EMBASE, CINAHL, Scopus, PsycINFO, Web of Science and Google Scholar between December 2023 and December 2024. Two reviewers independently screened titles, abstracts and full texts for inclusion and extracted data using Covidence software. Outcomes were tabulated according to measurement type and components of the WHO‐ICF framework.

**Main Contributions:**

A total of 658 titles and abstracts were screened; 255 progressed to full text review, with 49 full text articles being included. Twenty‐three studies (47%) followed a prospective observational design, 17 (35%) a retrospective case note review, 5 (10%) an experimental case control design, 2 (4%) survey design and 2 (4%) case reports. Studies included ILO [adults in 18 studies (37%), children 20 studies (41%), and both adults and children in 11 studies (22%)]. Outcomes were collected at two or more time points within a single day to over three years. Thirteen (26%) studies measured performance outcomes, 15 (31%) clinician‐reported outcomes, 48 (97%) patient‐reported outcomes, and 2 (4%) observer‐reported outcomes. Several of the studies considered the impairments of body function, structure and activity limitations in line with the WHO‐ICF framework but environmental factors and personal factors were rarely considered.

**Conclusions:**

The findings demonstrate variation in the outcome measures used in ILO research and that measures of personal and environmental impacts are rare. There is a need for consensus of a core outcome set across the spectrum of ILO research and/or clinics that capture the full range of impact and facilitate evidence synthesis.

**WHAT THIS PAPER ADDS:**

*What is already known on this subject*

- Inducible laryngeal obstruction is an upper airway disorder that causes physical, social and psychological impact on individuals. Outcome measures are used to assess progress or impact of an intervention.

*What this paper adds to the existing knowledge*

- Varied outcome measures were identified across studies, but there are many gaps in coverage, particularly in disease‐specific personal and environmental impact.

*What are the potential or actual clinical implications of this study?*

- This study identifies that there is heterogeneity in the current outcome measures used, and standardised measures are required that are disease‐specific and cover all domains of the World Health Organisation International Classification of Function, Disability and Health framework.

## 1 Introduction

Inducible laryngeal obstruction (ILO), also known as vocal cord dysfunction (VCD), is a transient, inappropriate and reversible narrowing of the laryngeal aperture typically in response to certain triggers (Halvorsen et al. 2017). The true prevalence of ILO is unknown, but it is more common in females (70%–80%) with a broad age range (Haines et al. 2018; Petrov 2019). Patients with ILO often have other comorbidities, both airway‐related (asthma, nasal disease, allergy and breathing pattern disorder), and non‐airway related (reflux, anxiety and neurological). It can both be misdiagnosed as asthma and co‐exist with asthma (Vertigan et al. 2021). The prevalence of ILO in asthma is reported as 20%–40%, although most reports are from specialist centres and comprise patients with more severe disease (Abdelwahab et al. 2020; Bardin et al. 2018). Eight million people in the United Kingdom have a diagnosis of asthma meaning a high proportion of the population will be living with ILO. The mainstay of treatment is speech and language therapy (SLT) but research on treatment efficacy is limited (Lee et al. 2018; Vertigan et al. 2006, 2020).

Diagnosing ILO is not straightforward, and in many patients the diagnosis has been overlooked for some time (Hull et al. 2016). Due to the transient nature of ILO symptoms, the consensus gold standard for diagnosis is direct visualisation of the larynx with laryngoscopy when symptomatic (Halvorsen et al. 2017; Christopher et al. 1983).

ILO is a distressing and transient condition (McConville and Thibeault 2024) and effects on the patient can be wide ranging (Ludlow et al. 2025). Episodes are often sudden and variable in severity (Fowler et al. 2015) with symptoms mostly localised to the throat or upper chest (Petrov 2019; Haines et al. 2018; Halvorsen et al. 2017). Key symptoms of ILO include dyspnoea, coughing, voice changes and throat tightness (Hull et al. 2016). Episodes are triggered by physical, environmental or psychological factors, for example, talking, laughing, exertion, strong scents, temperature changes or stress; although it can also occur without an identified stimulus (Marcinow et al. 2015; Anderson 2015). Individuals with ILO present across a variety of healthcare settings with differing levels of morbidity (Haines et al. 2018) which can lead to unnecessary medical treatment and high healthcare utilisation (Tiotiu et al. 2018; Abdelwahab et al. 2020).

We need to understand the impact of ILO on life and assess these effects to determine optimal treatment and management. The World Health Organisation International Classification of Function, Disability and Health (WHO‐ICF) framework provides a coherent view of different aspects of health from biological, individual and social perspectives (Stucki et al. 2002; WHO‐ICF 2001). The International Classification of Functioning, Disability and Health (ICF) focuses on two parts with five components. The two parts are “functioning and disability” and “contextual factors”. These and the five components are detailed below:

### 1.1 Part 1: Functioning and Disability

1. Impairment of body function: Impairment of the physiological function of the body system (including psychological function).
2. Impairment of body structure: Impairment of anatomical parts of the body such as organs, limbs and their components.
3. Activity limitations and participation restriction: Limitation in the ability to execute tasks at an individual level or to be involved in everyday life situations.

### 1.2 Part 2: Contextual Factors

1. Environmental factors: Refer to physical, social and attitudinal factors in the person's life and society which hinder or facilitate their functioning.
2. Personal factors: Refers to characteristics such as age, gender, ethnicity, personality, resilience or experiences.

The ICF framework underscores the importance of the interplay and influence of both internal and external factors to an individual's health status. The framework provides a standardised procedure that enables interventions and outcome measures to be mapped to it. Cieza et al., in 2002 and updated in 2005, produced rules such that meaningful concepts within each of the outcome measures are identified and linked to the most precise ICF category (Cieza et al. 2002; Cieza et al. 2005). Meaningful concepts are those that describe the health condition, person, functional activity or any of the environmental factors. For example, the Dyspnoea Index (DI) has two items; ‘Changes in weather affect my breathing problem’ and ‘My breathing problem causes me to restrict my personal and social life’; the concepts extracted from these items were weather changes and impaired social life (Gartner‐Schmidt et al. 2014). Weather changes were linked with the environmental factors and social life linked with participation.

There are ways of measuring impact on life. Clinical outcome assessments (COAs) are measures that describe how a patient feels, functions or survives (US Food and Drug Administration 2009), and provide justification for treatment on an individual patient level. When outcomes are measured and reported, they foster adoption of best practices, thus further improving outcomes (Pantaleon 2019).

Clinical outcome assessments are usually divided into four categories: 1) patient reported outcome (PRO) measures, 2) observer‐reported outcome (ObsRO) measures, 3) clinician‐reported outcome (ClinRO) measures and 4) performance outcome (PerfO) measures. Use of PRO measures is an essential aspect for improving clinical care, because they allow personalisation of treatment plans, enhance connections between patients and clinicians, and improve patient satisfaction (Chen et al. 2013; Pantaleon 2019). A PRO can be measured by self‐report or by interview provided that the interviewer records only the patient's response. Self‐report measures are typically captured in the form of a questionnaire or rating scale. Although self‐report measures are subjective in nature, they objectify a patient's perception. PROs can also assess the patient perspective on functioning or activities as observed by others. An ObsRO measurement is completed by a parent, caregiver or someone who regularly observes the patient in daily life. They are particularly useful for patients who cannot report for themselves. A ClinRO measurement is completed by a trained healthcare professional who uses clinical judgement to report on observed patient behaviours or signs. ClinRO measures cannot directly assess symptoms that are known only to the patient. ClinRO measures also include reports of clinical findings (e.g., laryngeal findings) or clinical events (e.g., asthma exacerbation) which can be based on clinical observations together with biomarker data, such as fractional exhaled nitric oxide (FeNO) and blood eosinophils in asthma. A PerfO measurement requires the patient to perform a set of movements or tasks. Scores for PerfO measures can be based on either an objective assessment (e.g., time to complete a task) or a qualitative assessment that is assigned a score (e.g., normal or abnormal mechanics). PerfO and PRO measures both capture the current state, but do not typically align with each other. PerfO measures tend to bring to light physiologic factors whereas PRO measures capture a patient's perception, beliefs, social factors and/or health factors (Bean et al. 2011).

Despite having ways of measuring impact of ILO on life we don't know if current studies use these methods consistently and if they cover measuring the full spectrum of function, disability and health in line with the WHO‐ICF framework.

### 1.3 Aims and Objectives

We aimed to systematically scope the literature which reports use of COA measures in individuals with ILO (children and adults), identifying what is measured and whether the measurements consider physical, social and emotional factors in line with the WHO‐ICF framework (WHO‐ICF 2001). A scoping review approach was chosen because the objective was to describe what is in the literature, rather than to compare different interventions.

## 2 METHODS

### 2.1 Defining Clinical Outcome Measures

For this review, a clinical outcome is a planned measurement made of an individual, group of people, or population which is attributable to an intervention or series of interventions (World Health Organisation 1998).

### 2.2 Design

This scoping review was conducted in accordance with the Preferred Reporting Items for Systematic Reviews and Meta‐Analysis for scoping reviews (PRISMA‐P) checklist and the PRISMA extension for scoping reviews (PRISMA‐ScR) (Tricco et al. 2018). The review was prospectively registered on the Open Science Framework database: https://doi.org/10.1111/1460‐6984.13007. It is part of a larger project developing and validating a PRO measure for ILO.

A preliminary search of MEDLINE, the Cochrane Database of Systematic Reviews and Joanne Briggs Institute (JBI) Evidence Synthesis was conducted, and no current systematic reviews or scoping reviews were identified on this topic. Over 1000 papers were found on the preliminary MEDLINE search with the search terms (‘inducible laryngeal dysfunction’ OR ‘Vocal Cord Dysfunction’) AND (‘measur*’ OR ‘outcome*’), so it was felt that there was sufficient evidence available to inform this review.

The scoping review was in keeping with the six‐stage scoping review framework described by Arskey and O'Malley and the revised recommendations by Levac et al. to allow methodological enhancement (Arskey and O'Malley 2005; Levac et al. 2010).

### 2.3 Research Questions

Through consultation with the research team and key stakeholders, the primary research question was defined as, ‘What outcomes are measured and documented in studies including people with ILO?’ The key stakeholders included patients living with ILO and health care professionals diagnosing and treating ILO. Two separate focus groups were completed where we discussed the aims and objectives of the study and key questions were developed. Our aim was to map measures recommended by existing reviews for use in children and adults with ILO and identify which areas were not being measured in line with the WHO‐ICF framework (WHO‐ICF 2001). Secondary research questions were:

1. Are different measures used depending on the patient group (adults and children)?
2. Are different measures used depending on the phenotype of ILO (classic ILO, lung‐associated ILO, exercise‐associated ILO and incident‐associated ILO)? (Leong et al. 2022).

### 2.4 Search Strategy

A systematic search was conducted of MEDLINE, EMBASE, CINAHL, Scopus, PsycINFO, Web of Science and Google Scholar. The Joanna Briggs Institute recommends using Population–Concept–Context (PCC) to develop strategies for scoping reviews (Peters et al. 2019), and the PCC format guided the development of our search strategy (table 1). The specific search strategy was adjusted to meet the requirements and format used within each database. The initial search was conducted between December 2023 and January 2024. The following search terms were used: ‘inducible laryngeal obstruction’ OR ‘ILO’ OR ‘vocal cord dysfunction’ OR ‘VCD’ OR ‘paradoxical vocal fold motion’ OR ‘PVFM’ AND ‘outcome’ OR ‘measure’ OR ‘measurement instrument’ OR ‘assessment’ OR ‘scale’ OR ‘questionnaire’. An updated search was completed in May 2024, December 2024 and March 2025, but no further studies were identified that met inclusion criteria. The set of included articles were those that met inclusion criteria and were also identified by searching reference lists of the articles and other literature.

**TABLE 1:** Framework for determining eligibility of research question.

P‐Population	ILO patients (adults and children)
C‐Concept	Health outcomes measured in research
C‐Context	Studies measuring health outcomes in ILO

### 2.5 Inclusion/Exclusion Criteria

Studies that met the following criteria were included in the review:

- Primary research studies published between 1983 and May 2024 [1983 was when the first literature that clearly described ILO (referred to then as vocal cord dysfunction) was published].
- Studies that report clinical outcomes measured in neonates, children or adults with ILO
- Studies that report outcomes measured by any medical professional working with ILO.
- Studies that report use of COAs (PRO, ObsRO, ClinRO or PerfO measures).
- Grey literature sourced from websites of respiratory medicine, universities, regulatory bodies, government educational departments, evaluations and working papers.

Studies were excluded from the review if they were:

- Published before 1983.
- Not written in English.
- Not available as full texts. Full texts that were not available through the University of Manchester or Manchester University NHS Foundation Trust electronic databases were requested through the librarians, but some papers were still unavailable as full texts.

Studies that discussed children and/ or adults were included to provide comprehensive coverage across the life‐course and to allow comparison across the different age groups. Single case studies were only included if they provided a detailed and unique example of a specific outcome measure, which added to the review and provided a diverse perspective. We excluded systematic reviews or scoping reviews that were not primary research and instead reviewed the original research to see if this should be included.

### 2.6 Study Screening

Identified studies were imported into Covidence (Covidence (2024) systematic review software, Veritas Health Innovation, Melbourne, Australia; available at https://www.covidence.org/.), which is a web‐based collaborative software platform that streamlines the production of systematic and other literature reviews. Duplicates were removed by Covidence during this process, and any further duplicates manually removed. The study screening was performed between December 2023 and January 2024. Updated searches were completed in May 2024, December 2024 and March 2025.

A three‐step process was followed for study selection. The main reviewers were SL and LJH. SF and LBD (supervisors of SL) were consulted if required, to discuss any conflicts. The following steps were then undertaken:

**Step 1**: Titles of articles retrieved through execution of the search strategy in each database were screened by SL. This reviewer applied the inclusion/exclusion criteria and recorded whether the article was being included or the primary reason for exclusion.
**Step 2**: Inclusion/exclusion criteria were applied to the abstracts of relevant articles identified in step 1 by SL using Covidence. Reviewers met at the end of the abstract screening process to discuss any disparities, referring to SF and LBD if required.
**Step 3**: SL and LJH worked independently to screen the full texts of each paper and either accepted, rejected or referred for discussion based upon inclusion/exclusion criteria. Any identified conflicts in the assessment were resolved through discussion between SL and LJH, consulting SF and LBD if required.

### 2.7 Data Extraction

Once the full text articles were agreed, data were extracted into a chart by the two main reviewers (SL and LJH). The chart contained study metrics (author, publication date), population characteristics, study aims and outcomes (table 2). The outcome measurements were classified into each of the WHO‐ICF components: body functions and structures, activities and participation, environmental factors and personal factors.

**TABLE 2:** Data charting of 49 included studies.

Author	Title	Aim	Study design	Sample size	Outcome measure	Time horizon of outcomes	Age range (years)	Phenotype	Key findings summary
Baxter et al. 2019 Australia	Multidisciplinary Team Clinic for VCD Directs Therapy and Significantly Reduces Healthcare Utilisation	Assess if MDT clinics for ILO improves health and reduces healthcare utilisation.	Prospective, observational, proof‐of‐concept study	80	GP visits, ED/ hospital admission, ACQ, ACT, Nijmegen, VCDQ, lung function, laryngoscopy, CT larynx,	12 months	57 ± 15; mean ± SD	ILO and ILO/asthma	Hospital attendances declined significantly in the 12 months following LR and there was a reduction in GP visits. There was a trend to improved BPD scores ILO detected by CT improved post LR. There were minimal changes on laryngoscopy post LR. There was significant reduction in healthcare utilisation post botox. There was reduction in ILO on laryngoscopy post botox. There were no changes in questionnaire scores post LR or botox.
Baxter et al. 2014 Australia	Abnormal Vocal Cord Movement Treated With Botulinum Toxin in Patients With Asthma Resistant to Optimised Management	Assess if treatment with botulinum toxin by local injection will benefit patients who have asthma, abnormal vocal Cord movement and intractable asthma symptoms.	Observational study	11	ACT, CT larynx, spirometry, oral and inhaled steroid use, patient self‐reporting.	Baseline, 1 and three months	60.9 ± 10.4	Abnormal vocal cord movement resistant to SLT	Improvements in ACT and CT larynx one month after botulinum injection. Changes on CT were not correlated with ACT scores. There were significant improvements in FEV1 and FVC. Steroid use was unchanged.
Campisi et al. 2019 Canada	Exercise‐Induced Laryngeal Obstruction: Quality Initiative to Improve Assessment and Management	The purpose of this project was to identify patient characteristics that can be uses to predict a higher likelihood of EILO and thus, streamline referrals for exercise‐endoscopy testing. This information would be used to improve the efficiency of the triage process and the timely access to exercise‐endoscopy testing.	Retrospective chart review	35 (13 follow up)	Physical activity levels (not clear how this is measured)	1–8 months	5–18 (mean age 14.1)	Paediatric EILO	Diagnosis of EILO had a negative impact on physical activity. All of the eight patients who were competitive athletes, seven became non‐active or recreationally active after their initial visit. All of the 35 patients who initiated an exercise endoscopy test, only 18 developed airways symptoms prompting endoscopy to visualise the cords. For the other 17 a definitive diagnosis of EILO could not be established because patients did not develop symptoms.
Chiang et al. 2013 USA	Exercise‐Induced Paradoxical Vocal Fold Motion Disorder: Diagnosis and Management	To review experience of diagnosis and treatment of EPVCMD.	Single institution retrospective review	67	Patient report of symptoms	Pre‐therapy and at each therapy session	18–81 (median age 45)	PVFMD and EPVFMD	48 patients (75%) reported either improvement or complete resolution of symptoms at their last follow up. The average number of LCT sessions was 2.2 in the EPVFMD and 2.5 in the PVFMD cohorts. Characteristic findings of EPVFMD in the resting patient while asymptomatic have been reported in the literature. 48% of patients had evidence of PVFMD on pre provocation laryngoscopy. 87% went on to develop EPVFMD with exertion. Laryngoscopy was not performed during exertion, but rather immediately following the reproduction of symptoms.
Christensen and Thomsen 2011 Denmark	Exercise‐Induced Laryngeal Obstruction: Prevalence and Symptoms in the General Public	Assess the prevalence and symptoms of EILOs and their relation to AHR.	Cross‐sectional study	237 step 1, 150 step 2, 98 step 3	Symptom questionnaire	12 months	14–24 (mean 18.26, median 18)	EILO and AHR	EILO was verified in day 2 in 42 participants (42.9%), AHR was diagnosed on day 1 in 23 (4.1%). There was no correlation found between AHR and the degree of laryngeal obstruction. None of the most commonly reported symptoms were specific for one of the diagnoses. No symptoms were specific for EILOs or AHR. Therefore, it was not possible to clinically differentiate between the two conditions
De Guzman et al. 2014 USA	Validation of the Dyspnoea Index in Adolescents With Exercise Induced Paradoxical Vocal Fold Motion	To validate the dyspnoea index quality of life instrument (previously validated for adults with breathing disorders) in children with EPVFMD and to determine the minimum significant DI change corresponding to patient reported or caregiver reported improvement or worsening of symptoms.	A longitudinal study	56	Adapted DI	Pre and post therapy	12–18	Paediatric EPVFMD	For 43 patients who perceived improvements, the mean decrease in the DI was 15.8 demonstrating a statistically significant change. Although validity was assessed only for the patients, a sole disagreement was found between patients and caregivers regarding global improvement
DeSilva et al. 2019 USA	Vocal Fold Botulinum Toxin Injection for Refractory Vocal Fold Motion Disorder	Demonstrate efficacy of vocal fold botulinum toxin injection for treatment of refractory paradoxical vocal fold motion disorder.	Retrospective review	13	DI, patient reported dyspnoea symptoms	Pre and post injection	16–73 (mean 40.69)	PVFMD resistant to SLT and LR	The mean number of injections was 3.85 (range 1–12). 11/13 patients experienced improvement in dyspnoea with 2/11 having complete resolution of symptoms after one injection. The two patients who did not have improvements eventually underwent tracheostomy.
Doshi and Weinberger 2006 USA	Long‐Term Outcome of Vocal Cord Dysfunction	To determine the long‐term outcome of ILO.	Retrospective review	49 (28 patients contacted)	Patient report of symptoms	Three years after diagnosis	EIVCD median age 14.9 (9–20), SVCD median age 13.5 (8–25)	Paediatric EIVCD, spontaneous occurring VCD	The long‐term outcome of VCD in our population demonstrated eventual resolution of symptoms in 26 of 28 patients, irrespective of whether they attended SLT or not.
Drake et al. 2017 USA	Functional Outcomes After Behavioural Treatment of Paradoxical Vocal Fold Motion in Adults	To identify patient perceptions of the impact of treatment for PVFM and characteristics associated with treatment outcomes.	Survey	39	Survey developed for this study. Questions related to their baseline symptoms and whether there had been changes in their health, voice or respiratory symptoms since treatment.	Six months post diagnosis. Median time after treatment 10 months.	18–32 (median 44.5)	PVFMD	43 /102 eligible participants responded. With regard to health status currently the most common response was ‘good’. More than two‐thirds of the respondents reported reduction in PVFMD anxiety and feelings of helplessness. Several of the participants (61%) reported an improvement in at least one activity, while the remainder (39%) reported no improvement for activity.
Fujiki et al. 2023 USA	Therapy Outcomes for Teenage Athletes With Exercise Induced Laryngeal Obstruction	To examine the outcomes of therapy with an SLP in addressing EILO symptoms in teenage athletes; including treatment gains reported from the course of therapy and the duration of these gains, the relationship between EILO symptoms and treatment and overall health related quality of life.	Prospective cohort study	59 (38 post therapy, 32 3 month and 27 6 month)	PedsQL, EILO survey (developed for this study)	Baseline, immediately post therapy, three and six months	≤18 (median 16.06)	Paediatric EILO	Immediately post therapy patients reported a significant reduction in times when they had to cease or limit physical activity because of their breathing problem. Patients continue to experience improvement, as they were involved in more intense and regular physical exertion over time. The PedsQL scores were lower in this population than would be expected at baseline. Therapy was not followed by any significant change in PedsQL score—even in patients who experienced a decrease in dyspnoea. PedsQL scores were still below normal range even when symptoms were controlled. Lower baseline scores did predict more frequent breathing problems 6 months post therapy.
Fujiki et al. 2024 USA	Examining Therapy Duration in Adults With Induced Laryngeal Obstruction	To identify factors predicting therapy duration and the likelihood of patients returning for additional therapy sessions following initial discharge.	Retrospective observational cohort design	350	If had a voice disorder (GRBAS), behavioural health diagnosis (yes/no), VHI, reports of having to reduce physical activity. Post therapy outcomes were from patient report online—no measurement	Baseline and post therapy	> 18	EILO/ ILO/ EILO+	Patients with EILO required significantly fewer sessions than those with ILO. Patients with a behavioural health diagnosis required significantly more sessions than those without such a diagnosis. Total VHI score significantly predicted number of therapy sessions. Patients with a voice complaint required significantly more sessions than those who did not have a voice complaint. Whether patients reported having to reduce physical activity due to EILO/ ILO symptoms predicted number of therapy sessions. On average, patients reported decreased physical activity required significantly more sessions prior to discharge.
Gallena et al. 2015 USA	The Effect of Exercise on Respiratory Resistance in Athletes With and Without Paradoxical Vocal Fold Motion Disorder	To investigate respiratory resistance as determined by airway perturbation device, exercise duration, and ratings of dyspnoea in athletes with and without ILO over repeated trials before and after rigorous, customised exercise challenge.	Group‐comparison study (one session only)	24 (12 EPVFMD, 12 control)	Modified BDS.	One minute intervals	median 14.5 (PVFMD), 14.8 (control)	Paediatric EPVFMD	On average the ILO group discontinued the exercise challenge test 1.9 min sooner than the control athletes. Dyspnoea ratings differed significantly between groups. As expected athletes with ILO exhibited a marked increase in Ri and a smaller but significant increase in Re immediately after exercise, suggesting biphasic ILO. For control athletes, Re decreased significantly immediately after exercise and stayed at a similar level after an additional minute of post‐exercise rest.
Gallena et al. 2019 USA	Short‐Term Intensive Therapy and Outcomes for Athletes With Paradoxical Vocal Fold Motion Disorder	To develop a treatment for athletes with PVFMD based on exercise physiology and learning theory principles and administer it over a pre‐established time frame.	Prospective, within subject, group design	11 (8 full sets)	Respiratory resistance using airflow perturbation device (APD), BDS, DI.	Pre‐treatment, post treatment and 6 weeks post treatment	12.4–17.6 years (mean 15.3)	Paediatric EPVFMD	After STI therapy athletes experienced an immediate improvement in Rr and perceived dyspnoea ratings. Participants who attended for extended follow up continued to experience physiological and perceptual improvement with their exercise breathing.
Gartner‐Schmidt et al. 2014 USA	Development and Validation of the Dyspnoea Index: A Severity Index for Upper Airway Related Dyspnoea	To develop and validate the dyspnoea index, quantify severity of symptoms in upper airway dyspnoea and validate the DI as an outcome measure.	Survey methodology	369 (200 in the development of the DI, 15 in cognitive interviewing, 51 participants and 57 healthy controls in the reliability and validity measures and 46 pre and post treatment).	DI	Pre and post treatment	13–82 (mean 49)	Upper airway respiratory disorder	Measures taken before and after behavioural, pharmacologic or surgical intervention showed a statistically significant decrease in scores. This showed that participants noticed an improvement in the severity of the dyspnoea symptoms or treatment.
Gaylord et al. 2020 USA	Struggling to Breathe: Inspiratory Muscle Training in Adolescent Athletes	To determine the efficacy of inspiratory muscle training (IMT) in controlling dyspnoeic episodes of adolescent athletes with symptoms of EILO.	Single subject experimental design	5	Modified BDS, DI, MPT, duration of running	Pre, during and post IMT	10–18	Paediatric EILO	According to visual and statistical analyses, IMT had a significant positive effect on perceived breathlessness in 3/5 participants. 3/5 participants demonstrated a significant MCID for reduced perceived breathlessness on the MBDS. The other 2/5 participants demonstrated a significant MCID for reduced perceived breathlessness in the follow up phase of this investigation. 2/5 participants demonstrated MPTs that were outside mean standard deviation for their age and gender. Both demonstrated improvements to within range post treatment. The other 3/5 were within range pre and post treatment showed improvements in IMT. The participants length of time for running did not show any consistent improvements. 4/5 participants showed a significant increase in DI scores. All participants reported significantly reduced use of their inhaler, event while continuing to participate regularly in sporting events.
Halevi‐Katz et al. 2019 Israel	Buteyko Breathing Technique for Exertion‐Induced Paradoxical Vocal Fold Motion	To determine the usefulness of buteyko breathing technique (BBT) to reducing dyspnoea in patients with EPVFM concomitant with hyperventilation.	Within subjects, repeated measures group design	15	DI, end‐tidal carbon dioxide (ETCO^2^), RTMV, HR	Initial evaluation (pre‐1), three weeks after pre one, 30 minutes prior to first therapy session (pre two), post therapy (post one) and six weeks after last therapy session (post two).	14–68 (mean 28)	EPVFMD and hyperventilation	All of the 15 patients who were enrolled in the study, three participants were lost to attrition before treatment began. 10/12 participants completed the protocol in its entirety. There was a statistically significant decrease in DI scores over time. Post hoc analysis showed a significant decrease in DI score pre and post treatment. RTMC reduced after BBT. The RTMV measurements showed a statistically significant decrease over time. Changes in HRs were not statistically significant across the four time points. Mean ETCO2 post hoc analysis indicated that the increase was not statistically significant between any of the time points.
Hatzelis et al. 2012 USA	Paradoxical Vocal Fold Motion: Respiratory Retraining to Manage Long‐Term Symptoms	The objective of this study was to determine if respiratory retraining exercise alone would eliminate her breathing difficulties.	Case report	1	Spirometry pre and post, patient symptom severity rating scale 1–5. Laryngoscopy	Diagnosis, post treatment, one month post treatment, three months post treatment.	24	EPVFMD	PVFMD is an elusive diagnosis that can be easily overlooked, misdiagnosed, or otherwise treated inappropriately. The multitude of symptoms, aetiologies and their interactions associated with PVFM, including shortness of breath, chronic cough and reflux all exacerbate the difficulty of properly diagnosing the condition.
Irewall et al. 2023 Sweden	A Longitudinal Follow‐up of Continuous Laryngoscopy During Exercise Test Scores in Athletes Irrespective of Laryngeal Obstruction, Respiratory Symptoms and Intervention	This study aimed to compare CLE scores in athletes over time, irrespective of respiratory symptoms and grade of laryngeal obstruction.	Prospective study	98 (only 29 follow up)	Spirometry, eucapnic voluntary hyperventilation, CLE test, European Community Respiratory Health Survey II (ECHRS II).	Baseline and six months post	16–27	EILO	CLE scores mainly remained stable over time. Twenty‐seven per cent of those with moderate supraglottic obstruction at baseline and given advice on breathing techniques had mild obstruction at follow up. Seventeen per cent with no‐to‐mild supraglottic obstruction at baseline had moderate obstruction at follow up.
Ivancic et al. 2021 USA	Reduced Asthma Medication Use After Treatment of Paediatric Paradoxical Vocal Fold Motion Disorder	To determine whether the diagnosis and treatment of PVFMD leads to decreased asthma medication use and to determine dyspnoea outcomes following diagnosis and treatment for PVFMD.	Prospective observational study	26	Medication questionnaire, DI—adapted to grade mild, moderate, severe.	Initial, start of first return visit, three3 months, after six months.	11–17 (mean 14)	Paediatric PVFMD	Seventy‐three per cent of the patients in this study had previously been diagnosed with asthma. Forty‐six per cent reported relief from inhalers. Many reported relief was incomplete. The diagnosis and treatment of PVFMD demonstrated a downtrend in asthma medication use from diagnosis to last follow up. A significant decrease in mean asthma medication use was seen only in the full therapy group at last follow up.
Kramer et al. 2017 USA	Does Treatment of Paradoxical Vocal Fold Movement Disorder Decrease Asthma Medication Use	To determine whether diagnosis and treatment of PVFMD leads to decreased asthma medication use. Secondary objectives include determining initial rate of asthma medication use, characterising symptom improvement and correlating with PFTs.	Prospective observational study	66	Medication questionnaires, PFTs and subjective symptom improvement	Initial, therapy session 1, six months	13–80 (median 42)	PVFMD	The mean asthma medication score decreased from 4.85 at diagnosis to 2.40 at last follow up. The mean decrease in asthma medication score was 2.68 for patients completing a full therapy course, 2.60 for patients completing a partial therapy course and 3.44 for patients completing no therapy. The mean asthma medication score decreased from 4.62 to 3.29 after patients received a diagnosis alone, before any treatment. 56 (84.8%) were using asthma medications at the time of presentation. 54 (82%) reported symptomatic improvement of their symptoms.
Kumeresan et al. 2023 USA	Predictors of Voice Therapy Efficacy in Vocal Cord Dysfunction at a Tertiary Care Centre	To determine if there is an association between VCD and patient demographic and clinical factors and to evaluate the role of voice therapy in the treatment of VCD.	Retrospective chart review	184	Patient self‐reported percentage improvement of VCD symptoms	Beginning of each voice therapy session	7–72 (mean 16± 7)	VCD	The mean number of voice therapy sessions was 2.2 ± 1.5. Of 107 patients (58.2%) patients with documented breathing improvement were 72.5 ± 21.5%. This study suggests clinical implications for the use of voice therapy as a treatment for VCD. Patients reported symptom improvement that positively correlated with the number of voice therapy sessions.
LeBlanc et al. 2021 USA	Visual Biofeedback for Paradoxical Vocal Fold Motion	To determine a change in DI scores pre and post intervention 3 month follow up. To measure change in asthma medication, use from baseline to follow up.	Prospective, non‐randomised, non‐comparative clinical study	34	DI	Initial visit and each follow up	17‐67 (average 36.9± 14.1)	PVFMD	All of the 25 patients, 17 completed the dyspnoea index questionnaire pre and 3 months post intervention, with statistically significant reduction in scores and a mean difference of 12.1.
Lunga et al. 2022 USA	Economic Burden Associated With Management of Paradoxical Vocal Fold Motion Disorder	To estimate associated pre and post diagnosis direct and indirect healthcare cost, and to compare the cost of post diagnosis care among patients who did and did not undergo standard of care speech therapy.	Retrospective chart review	110	Patient reported successful or unsuccessful	Baseline and 12 months post	Median age 52 (IQR 32–62)	PVFMD	Patients who completed the recommended course of treatment (*n* = 53) had a median cost of care of $3,450 in the first post diagnosis year. Treatment was unsuccessful in 85% of those who completed therapy. Successful treatment (*n* = 45) was associated with $9,199 lower total cost compared to those whose therapy was unsuccessful.
Maat et al. 2011 Norway	Exercise‐Induced Laryngeal Obstruction: Natural History and Effect of Surgical Treatment	To assess the natural course of the supraglottic type of EILO in the patients diagnosed on the CLE test and to assess the long‐term effects from the surgical treatment of supraglottic EILO.	Retrospective, observational, longitudinal, follow‐up study	94 (23 ST, 71 CT)	VAS and CLE	Pre and post treatment	CT 14.7 (3.7), ST 15.1 (3.8)	Paediatric EILO	Symptoms and laryngeal function improved slightly in the patients treated with information alone, but surgically treated (ST) patients with supraglottic obstruction induced by exercise was significantly more effective measured at both the glottic and supraglottic level.
Marcinow et al. 2014 USA	Paradoxical Vocal Fold Motion Disorder in the Elite Athlete: Experience at a Large Division I University	To review the diagnosis and management of paradoxical vocal fold motion disorder in elite athletes.	A single institution retrospective review	46	Patient rating of their symptoms after each therapy session, no improvement, partial improvement or complete resolution.	After each SLT session	21 (18–69)	PVFMD	All of the patients who completed 1 session, three reported improvement or complete resolution of symptoms. Ten patients who completed two therapy sessions reported improvement or complete resolution of symptoms. Nighty per cent of the patients who attended 3 therapy sessions reported improvement or resolution of symptoms. All of the patients who attended four or more LCT sessions reported improvement or resolution of symptoms.
Marcinow et al. 2015 USA	Irritant‐Induced Paradoxical Vocal Fold Motion Disorder: Diagnosis and Management	To review outcomes of the diagnosis and treatment of irritant induced vocal fold motion disorder.	Retrospective chart review	34 (Irritant PVFMD), 76 (PVFMD)	Patient rating of their symptoms after each therapy session, no improvement, partial improvement or complete resolution.	After each SLT session	Median 46 (27–73)	Irritant induced PVFMD	LCT was well tolerated, and all patients who attended >/ = 2 sessions reported subjective reduction or complete elimination of dyspnoea symptoms and improved tolerance to offending odours. The rates of improvement following >/ = 2 sessions of LCT were similar in comparison with patients with vocal fold constriction noted following breath holding and counting tasks (100%) and with patients with vocal fold constriction noted following odour testing (100%). In comparison, patients in our non IPVFMD cohort had lower rates of improvement with LCT: 82% of patients with non‐IPVFM showed improvement or complete resolution of symptoms with >/ = sessions. The rates of improvement in the non‐IPVFMD cohort following 1 session were similarly low at only 23%.
Mathers‐Schmidt et al. 2005 USA	Inspiratory Muscle Training in Exercise Induced Paradoxical Vocal Fold Motion	To determine if inspiratory muscle training would result in increased inspiratory muscle strength, reduced perception of exertional dyspnoea, and improved measures of maximal exercise in an athlete with EPVFMD.	Within subject withdrawal design study	1	Spirometry pre and post, laryngoscopy pre and post, maximum inspiratory pressure (MIP) measurements, dyspnoea ratings during the symptom trigger level of activity	Baseline, end of third week of phase 1 IMT, end of fifth week of phase 1 IMT, end of third week of phase 2 IMT, end of fifth week of phase 2 IMT.	18	EPVFMD	MIP increased substantially across both IMT treatment phases. There was essentially no change in MIP after the no treatment period. By the end of the IMT withdrawal phase, the MIP was 102.8 ± 1.8% when compared with the final MIP of the proceeding treatment phase. During exercise that previously triggered PVFM symptoms, participant ratings decreased after IMT. There was no change in dyspnoea rating after the IMT withdrawal phase. The post laryngoscopy showed no evidence of vocal fold adduction after a period of exercise
Maturo et al. 2011 USA	Paediatric Paradoxical Vocal‐Fold Motion: Presentation and Natural History	To describe (1) a cohort of children with PVFM who were referred to a multidisciplinary airway centre and (2) the outcomes of various treatment modalities including SLT, reflux and psychiatric treatment.	Chart review	59	Subjective symptom resolution and/or return to activity	Pre and post therapy	13.64 (8–18)	Paediatric PVFMD	Speech therapy had a high success rate in patients who presented with symptoms only during exercise. Speech therapy was not as successful for patients who presented with symptoms at rest only. The majority of patients were not found to have vocal fold adduction on inspiration during examination
Murry et al. 2006 USA	Respiratory Retraining Therapy and Management of Laryngopharyngeal Reflux in the Treatment of Patients With Cough and Paradoxical Vocal Fold Movement Disorder	To describe the outcome of patients with cough and PVFMD treated with respiratory retraining therapy and management of LPR.	Prospective study	30 (20 patients with PVFMD and 10 controls)	Subjective rating of cough, PFTs and RSI	Pre and post therapy	Mean 54 (18–87)	PVFMD	All patients related subjective improvement in their symptoms of 6.4 scale points. The mean, FIV0.5/ FIVC pre and post treatment showed a significant difference to the control group. There was no significant difference between the two groups with respect to FVC or FEV1.0/FIVC. There was a significant improvement in the RSI pre‐treatment mean value of 25.2 to posttreatment mean value 12.6.
Nacci et al. 2011 Italy	Respiratory Retraining Therapy in Long‐Term Treatment of Paradoxical Vocal Fold Dysfunction	To evaluate the long‐term respiratory retraining therapy in cases of PVFMD	Chart review	20 (10 group A, 10 group B)	Subjective rating of the degree of dyspnoea	The day before the first session of therapy (T0), on the day of the first therapy session 12 months after the first one (T1), on the day of the first therapy session 24 months after the first 1 (T2)	44.2 ± 9.6	PVFMD	After one year of treatment (T1) both groups showed improvement in the severity scale and in the number of episodes as compared to T0. When the groups were compared (group A, group B0 there was greater improvement in the severity scale and in the number of episodes in group B, the one undergoing the higher number of cycles of therapy.
Norlander et al. 2015 Sweden	Surgical Treatment is Effective in Severe Cases of Exercise Induced Laryngeal Obstruction: A Follow‐up Study	To investigate how symptoms and level of physical activity change over time in patients with EILO who have undergone surgery, patients with EILO treated conservatively and patients testing negatively for laryngeal obstruction at CLE test.	Observational, longitudinal follow up study	84	Questionnaire about breathing difficulties and symptom severity VAS	pre‐CLE and 1–3 years post	16.5 (15.0–17.6)	EILO	The group of surgically treated EILO subjects reported decreased breathing problems at follow up when assessed by changes in VAS. In contract, the conservatively treated and subjects who tested negative had not improved. In addition, none of the surgically treated reported exercise induced dyspnoea as a cause of decreased or adjusted physical activity while amongst the conservatively treated EILO subjects, 41.2% of them reported less activity or adjusted physical activity due to exercise induced dyspnoea at follow up.
Novakovic et al. 2021 Australia	Supraglottic Botulinum Toxin Improves Symptoms in Patients With Laryngeal Sensory Dysfunction Manifesting as Abnormal Throat Sensation and/ or Chronic Refractory Cough	To 1) Describe the clinical characteristics of laryngeal sensory dysfunction in a cohort of patients referred for chronic refractory cough and abnormal throat sensation. 2) Describe a new treatment of supraglottic laryngeal botulinum toxin in the symptomatic management of laryngeal sensory dysfunction. 3) Evaluate the efficacy of using botulinum toxin A in treatment of a pilot group of patients presenting with different phenotypes.	Retrospective review	14	LHQ, CSI, RSI, VHI‐10	Pre and within 3 months post treatment	48.7 (32–76)	LSD	Following botulinum toxin there were statistically significant improvements in the primary patient reported outcomes of LHQ and CSI. Despite RSI being developed as a tool for LPR symptoms, there is a lack of agreement between its score and laryngopharyngeal pH monitoring. Sub‐item analysis showed significant Improvement in the items relating to abnormal sensation, excess throat mucous or post nasal drip and sensation of something sticking in the throat which are all symptoms common to LSD. The CSI scores decreased significantly after supraglottic BTS injection, supporting its role as a potential treatment for CRC. The VHI‐10 scores did not change significantly despite the common reports of voice change after treatment.
Oda et al. 2022 Japan	Vocal Cord Dysfunction Detected by a Three‐Dimensional Image of Dynamic Change in Respiratory Resistance in a Patient with Difficult to Treat Asthma: A Case Report	To discuss a case where MostGraph was used to evaluate the physiological changes caused by VCD and asthma.	Case report	1	Forced oscillation technique (FOT) measuring respiratory impedance, ACQ‐5	Pre and post treatment	74	VCD	Initially in the inspiratory phase, the respiratory resistance (Rrs) at 5 and 20 Hz, resonance frequency and area of reactance (ALX) were remarkably high, while the respiratory reactance (Xs) was low, especially in the inspiratory phase. 3D imaging showed increased spikes in Rrs in the inspiratory phase. On starting speech therapy, the symptoms gradually improved, as indicated by a reduction in ACQ‐5 and disappearance of inspiratory spikes.
Olin et al. 2022 USA	Development and Validation of the Exercise‐Induced Laryngeal Obstruction Index (EILODI)	To develop, validate and define minimal clinically important difference for a PROM to be used with adolescents and young adults with EILO.	Longitudinal study	219	EILODI	Days 0, 14 and 28	15.3 ± 1.9 years (EILO), 16.4 ± 3.6 (controls)	EILO	After a process of item reduction, a 12‐item metric with a total score ranging from 0–48 was developed. Mean scores of patients with EILO and healthy controls at baseline were 28.8 ± 7.4 and 4.5 ± 7.4 retrospectively. A MCID of 6 was determined by comparison of index change with changes in categorical self‐assessments of improvement.
Rameau et al. 2012 USA	Multidisciplinary Approach to Vocal Cord Dysfunction Diagnosis and Treatment in One Single Session: A Single Institutional Outcome Study	To evaluate whether patient education and behavioural intervention in the same session that ILO is diagnosed provides long term therapeutic benefits in children.	Retrospective review	22	Subjective questionnaire	Pre and post treatment	13.4 (7–20)	ILO	Nineteen patients (86.4%) reported improvement of ILO symptoms in frequency and/ or severity. The three who continued to experience significant symptoms presented with complex clinical pictures. Following treatment, 11 patients were able to increase their previous levels of activity and 10 patients were able to maintain their previous level of activity. Of the 15 patients with co‐existing asthma, four patients reported a decrease in inhaler use following clinic visit.
Richards‐Mauze and Banez 2014 USA	Vocal Cord Dysfunction: Evaluation of a Four‐Session Cognitive Behavioural Intervention	To examine the impact of a four‐session cognitive‐behavioural intervention to assess adolescents' ratings of symptom management and functional disability of VCD.	Observational study	36	Subjective VCD specific rating scales, the Health Locus of Control (CHLC) questionnaire, the Functional Disability Inventory (FDI), the Functional Disability Inventory (FDI)—Parent report.	VCD specific rating scales—pre therapy and during each therapy session. CHLC, DDI—pre and post therapy	14.80 ± 2.08	VCD	Results suggested significant decrease in symptom severity and functional impairment as well as significant improvement in perceived coping. Paired sample t‐tests also showed significant improvement post intervention. Both children and parent reported decreased functional disability scores postintervention. Changes in children's self‐reported health related locus of control were not significant on any subscales.
Sandnes et al. 2022 Norway	Clinical Responses Following Inspiratory Muscle Training in Exercise Induced Laryngeal Obstruction	To assess laryngeal outcomes shortly after IMT, and to compare self‐reported symptoms with a control group 4–6 years later.	A register based descriptive follow up study	116 (58 IBA + IMT, 58 IBA only	IBA+IMT—CLE, questionnaire, IBA only questionnaire	CLE—diagnosis and 2–4 weeks after. Questionnaire—baseline and 4–6 years post diagnosis	IBA+IMT 17.5 (10–30), IBA 15.2 (12–21)	EILO	Self‐reported symptoms and laryngeal findings had improved in most participants shortly after treatment with standardised IBA plus six weeks of IMT. Symptom improvement was associated with improved CLE score, particularly the glottic level. After 4–6 years, self‐reported symptoms had improved to similar levels both in the IBA+IMT group and IBA‐group. Symptom resolution was rare, and respiratory problems still disturbed most participants during exercise. The level of physical activity had decreased in most participants during the 4–6 year follow‐up.
Sandnes et al. 2019a Norway	Severe Exercise Induced Laryngeal Obstruction Treated with Supraglottoplasty	To investigate efficacy and safety of laser supraglottoplasty as treatment for severe supraglottic EILO.	Retrospective chart review	45	CLE, symptom scores, lung function, distance completed on treadmill, VE, peak VO_2_, MHR and MVV, RR at peak exercise	Pre‐ and post‐surgery	15.9 (10–25)	Supraglottic EILO	All patients had significantly lower CLE scores after surgery, with sum‐score significantly reduced from 5.38 to 2.36; most improvements explained by reduced supraglottic scores at maximum exercise. In 21/45 patients, glottic obstruction also decreased at maximum intensity exercise. After surgery perceived subjective symptoms improved in 38/44 (86%) patients. Lung function, distance completed on treadmill, VE, peak V02 and MHR did not differ after surgery. MVV had increased significantly after surgery, and RR at peak exercise was reduced.
Sandnes et al. 2019b Norway	Exercise‐Induced Laryngeal Obstruction in Athletes Treated With Inspiratory Muscle Training	To explore effects from Inspiratory Muscle Training used in athletes with EILO.	Explorative study design	28	CLE, lung function, ergo spirometry, symptom scores	Pre and post treatment	16.4 (12–25)	EILO	After a six‐week training period with IMT, most athletes (79%) reported subjective improvement. The CLE scores improved in 82% had become normal in 18% and worsened in 7% (2 patients). Improvements were linked to a change at the glottic laryngeal level at peak exercise. Ergo spirometric variables revealed significantly higher VE but were otherwise unchanged.
Schonmman et al. 2023 USA	Multi‐Institutional Study of Patient Reported Outcomes of Paradoxical Vocal Fold Motion	To explore patient reported outcome measures of paediatric paradoxical vocal fold motion through a multi‐institutional study of geographically diverse United States medical facilities to assess long term management and outcomes.	Observational study	65	Survey developed for this study.	Post treatment	12.9	Paediatric PVFMD	Only five treatments were considered effective by the majority of the participants. The treatments that participants tried most often were breathing exercises (89.2%), bronchodilator treatments (45%) and allergy medications (35.4%). Two‐thirds (66%) of all participants who tried breathing exercises reported them effective. Whilst 40% of participants experienced a resolution of their PVFMD symptoms within three months of diagnosis, almost one quarter (23.3%) of participants did not experience resolution until greater than one year after diagnosis. While otolaryngologists consider biofeedback another mainstay of treatment, it was rarely recalled by participants and judged as effective by just one half of those who tried it.
Shembel et al. 2019 USA	Perceptual Clinical Features in Exercise Induced Laryngeal Obstruction: Toward Improved Diagnostic Approaches	To identify patient centred perception of symptoms that could distinguish adolescent athletes with EILO from athletes without at baseline (rest) and during an exercise challenge (provocation) and to quantify symptom severities for use as a preliminary diagnostic benchmark.	Observational study	27 (13 EILO, 14 control)	Severity ratings (0–100), EATQ‐R, heart rate monitor, blood pressure, leg fatigue parameter	Baseline, during laryngoscopy without exertion, maximum exertion (laryngoscopy with exercise challenge)	14.46 (1.94) for EILO, 16.87 (1.19) for control group	EILO and control group	Participants in the EILO group reported greater inspiratory and expiratory dyspnoea than the control group in both baseline and exercise conditions, The group differences were statistically significant with exercise but not at baseline with inspiratory dyspnoea and both at rest and with exercise with expiratory dyspnoea. Average severity levels of self‐reported stridor both at baseline and during exercise were similar between groups. On average participants in the EILO group reported more throat tightness than the control group both at baseline and exercise. Results on leg fatigue ratings showed the two groups perceived comparable levels of exertion at both baseline and exercise.
Strojanovic et al. 2022 Australia	Diagnostic and Therapeutic Outcomes Following Systematic Assessment of Patients With Concurrent Suspected Vocal Cord Dysfunction and Asthma	To evaluate diagnostic and therapeutic outcomes following systematic assessment for patients with concurrent suspected VCD and asthma.	Observational study	212	VCDQ, DI, LHQ, and subjective reporting of symptoms	Baseline and visit 2	47 ± 11.3, range 17–82	VCD, VCD and asthma, asthma, no asthma or VCD	Sixty‐six of 90 (73%) patients with objective confirmation of VCD who attended follow up reported a subjective improvement in laryngeal symptoms. Patients who received two or more sessions of speech pathology were more likely to reported subjective improvement in laryngeal symptoms than those who received one or no session of speech pathology. Among those who received two or more sessions of speech pathology there was no improvement in questionnaire scores, whether by VCDQ, LHQ, DI. No significant difference in scores was seen between those who received two or more sessions of speech pathology and those who did not.
Sullivan et al. 2001 USA	A Treatment for Vocal Cord Dysfunction in Female Athletes: An Outcome Study	To describe the outcome of a speech pathology treatment program for vocal cord dysfunction.	A retrospective, non‐randomised group design	20	Survey	Six months post treatment	14.1 (12–17)	Adolescent females with EIVCD	Nineteen of 20 participants (95%) reported the ability to control symptoms of VCD using abdominal breathing techniques used in the intervention session. Thirteen participants (65%) reported no VCD episodes during the previous six months. One participant (5%) reported continued frequent episodes of VCD with increased episodes outside treatment. All of the participants reported continue athletic participation. Sixteen participants (80%) reported no use of asthma medications to control symptoms of VCD during exercise. Four participants (20%) indicated that they occasionally use the asthma medications before exercise but didn't know if they really need it.
Traister et al. 2016 USA	The Morbidity and Cost of VCD Misdiagnosed as Asthma	To assess if current measures of asthma control contribute to the findings of ILO being misdiagnosed as asthma and if a simple set of breathing exercises would be an effective low‐cost treatment option for those with ILO.	Retrospective analysis	89	ACQ, subjective outcomes	Pre and post treatment	47 (37–56)	VCD	The effectiveness of breathing exercises did not differ between the different VCD groups. We further quantified the number of VCD symptomatic episodes in 15 subjects before and after providing medical treatment and breathing exercises. After a two month follow‐up, these subjects reported a significant decrease in monthly episodes after medical treatment. The average ACQ score in subjects with VCD misdiagnosed with asthma was 1.58, whereas those with uncontrolled asthma had an average score of 3.05.
Vance et al. 2021 USA	Paradoxical Vocal Fold Motion: A Retrospective Analysis	To determine characteristics of patients with confirmed PVFM and to evaluate efficacy of current treatments.	Retrospective analysis	40	DI and subjective rating	Before and after treatment	30.25	PVFMD	80% of patients underwent voice therapy (VT), 93% of patients underwent LPR therapy, 30% underwent psychotherapy and 23% underwent botulinum toxin (BT) treatment. Patients who received BT, VT and LPR treatment had a 90% subjective improvement rate. Patients who received VT and LPR treatment had a 43% subjective improvement rate. The clinical improvement rate was statistically significant. There was no statistical difference between VT and LPR combined versus VT or LPR treatment alone.
Vertigan et al. 2014 Australia	Development and Validation of the Newcastle Laryngeal hypersensitivity questionnaire	To develop a laryngeal hypersensitivity questionnaire for patients with laryngeal dysfunction syndromes in order to measure the laryngeal sensory disturbance occurring in these conditions.	Longitudinal study	97	LHQ	Ppre and post treatment	56	Laryngeal hypersensitivity and control group	The mean pre‐ and post‐treatment improvement in the Newcastle Laryngeal Hypersensitivity Questionnaire scores was 2.3 (SD 3.5). Change scores also improved significantly for each factor, obstruction 0.9, irritation 0.9, pain/thermal 0.5. The MCID for the total score was calculated at 1.75.
Warnes and Allen 2005 USA	Biofeedback Treatment of Paradoxical Vocal Fold Motion and Respiratory Distress in an Adolescent Girl	To evaluate the effectiveness of surface electromyography (EMG) biofeedback to treat paradoxical vocal old motion.	A changing criterion design	1	Pain VAS, adaptive functioning VAS—both patient and mother	Pain measures—pre‐treat and 4 x daily. Adaptive functioning before and after treatment	16	Paediatric PVFMD	EMG was found to be an effective means of gaining control over muscle tension near the vocal cords. Overall baseline tension levels were reduced over 60%. There were also corresponding reductions in episodes of respiratory distress and chest pain. Prior to biofeedback training, the participant reported missing 25% of all school days due to distress and pain. Following the sixth session, no school absences due to PVFMD symptoms were reported. The mother reported marked reduction in interference of daily functioning by PVFMD symptoms from baseline to treatment.
Yibrehu et al. 2020 USA	Outcomes of Paradoxical Vocal Cord Motion Diagnosed in Childhood	To explore long term patient reported outcome measures of paediatric paradoxical vocal cord motion including ease of diagnosis, management, symptom duration and effect on quality of life.	Observational study	18	Survey developed for this study	Post treatment	15	Paediatric PVFMD	Ongoing symptoms were reported in 13/18 participants (72%). Nearly two‐thirds of participants using lifestyle modifications for PVFMD management reported them to be effective, while most using biofeedback considered it minimally effective. 56% of participants reported breathing exercises as an effective tool.
Zalvan et al. 2019 USA	A Trigger Reduction Approach to Treatment of Paradoxical Vocal Fold Motion Disorder in the Paediatric Population	To characterise the typical patient diagnosed with PVFMD and analyse the change, or lack thereof, in their subjective scores upon presentation and following treatment with our trigger reduction approach.	Retrospective chart review	24	VHI‐10, RSI, DI, CSI.	Pre and post treatment	15 (10–17)	Paediatric PVFMD	Of the four outcome measures, only a change in the DI met statistical significance. Of 24 patients, 18 demonstrated a reduction in DI following the trigger reduction protocol. Using reduction in DI as a continuous variable to assess response, the patient cohort experienced a 4.62 (95% confidence interval) mean point reduction. In contrast, changes in CSI, RSI and VHI did not meet statistical significance.

**Abbreviations**. ACQ—Asthma; ACQ—Asthma Control Questionnaire, ACQ‐5—Asthma Control Questionnaire (5 questions), ACT—Asthma Control Test, VCDQ—Vocal Cord Dysfunction Questionnaire, AHR—airway hyperresponsiveness, BDS—Borg dyspnoea scale, CLE—continuous laryngoscopy during exercise, CSI—cough severity index, DI—dyspnoea index, EATQ‐R—early adolescent temperament questionnaire, EPVFMD—exercise paradoxical vocal fold motion disorder, EILO—exercise induced laryngeal dysfunction cord dysfunction, HR—heart rate, ILO—inducible laryngeal dysfunction, LCT—laryngeal control therapy, LHQ—laryngeal hypersensitivity questionnaire, LR—laryngeal retraining, LPR—laryngo‐pharyngeal reflux disease, MCID—minimal clinical important difference, MHR—maximal heart rate, MPT—maximum phonation time, MVV—maximal voluntary ventilation, PedsQL—Paediatric Quality of Life inventory, PFTs—pulmonary function tests, PROMs—patient reported outcome measures, PVFMD—paradoxical vocal fold motion disorder, RR—respiratory rate, RSI—reflux symptom index, RTMV—respiratory tidal minute volume, SLP—speech and language pathologist, SLT—speech and language therapist, VAS—visual analogue score, VCD—vocal cord dysfunction, VCDQ—Vocal Cord Dysfunction Questionnaire, VE—minute ventilation, VHI‐10—voice handicap index, VO_2_—maximum rate of oxygen.

*VCD, PVFMD, ILO, EPVFMD, EILO, SLT and SLP are used interchangeably in the table depending on the terminology used in the study.

ICF linking rules were used in this study, such that meaningful concepts within each of the outcome measures are identified and linked to the most precise ICF category (Cieza et al. 2005). Several outcome measures linked with more than one ICF category. Both main reviewers (SL and LJH) independently rated which impairments had been considered and resolved any conflicts through discussion.

### 2.8 Analysis and Synthesis

Outcomes were initially sorted according to domains: PROs (survey, questionnaire or scale), PerfOs, ClinROs, ObsROs and time horizon of measurement. Responses were then sorted into subcategories. Quality assessment was not used consistent with scoping methods (Peters et al. 2015).

### 2.9 Patient and Public Involvement

Our patient partner (LS) was engaged throughout the study as a consultant and knowledge user. Specifically, she provided input regarding the search strategy and grey literature search. The search strategy and summary of each stage of study screening was discussed with LS at monthly meetings. At this time, the patient partner reflected on lived experience to ensure that any amendments to the search strategy and inclusion/exclusion criteria supported a patient‐centred understanding of health outcome measurements. In addition, LS will be involved in the dissemination of findings connecting with patient networks to provide a summary of results. LS is participating in the authorship and development of all manuscripts related to this research.

## 3 Results

### 3.1 Study Selection

**FIGURE 1:**
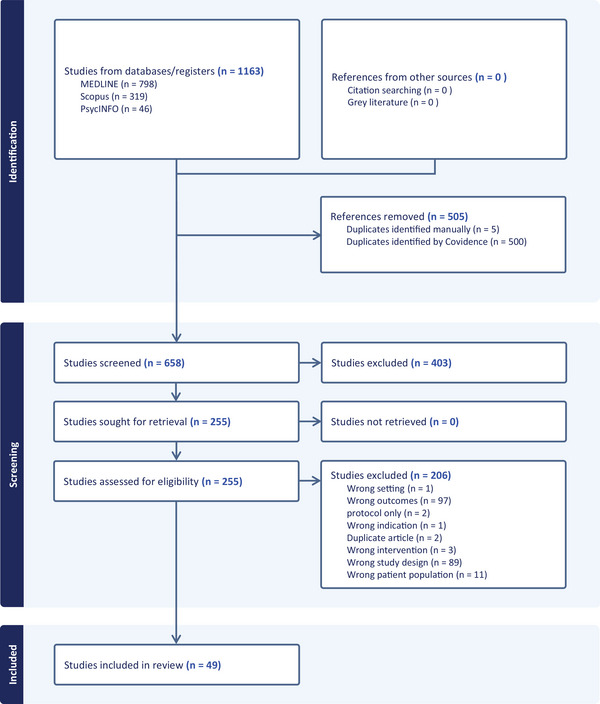
PRISMA flow.

Following duplicate removal, 658 titles and abstracts were screened; 255 progressed to full text review with 206 not meeting the inclusion criteria. The remaining 49 studies were included in the review. See PRISMA flow diagram (Figure 1) for details and reasons for exclusion.

### 3.2 Description of Included Studies

#### 3.2.1 Time and Place

Table 3 summarises the characteristics of included studies. The median interquartile range (IQR) year of publication was 2017 (2014–2021). Studies were undertaken in nine countries, most commonly the USA (*n* = 33) and Australia (*n* = 5).

**TABLE 3:** Description of included studies.

Studies (*n* = 49)	*N*	%
USA	33	67
Australia	5	10
Norway	4	9
Sweden	2	4
Canada	1	2
Denmark	1	2
Israel	1	2
Japan	1	2
Italy	1	2
**Study design (*n* = 49)**		
Observational design	23	47
Retrospective review	17	35
Experimental design	5	10
Survey design	2	4
Case reports	2	4
**Age group (*n* = 49)**		
Children	20	41
Adults	18	37
Children and adults	11	22
**Phenotype (*n* = 49)**		
ILO	27	55
E‐ILO	20	41
ILO and E‐ILO	2	4

#### 3.2.2 Design and Sample

Out of the 49 studies, 23 (47%) followed a prospective observational design, 17 (35%) were retrospective case note reviews, five (10%) an experimental case control design, two (4%) survey design and two (4%) case reports. The phenotypes included: adult ILO, 18 studies (37%), of which 14 (29%) studied ILO, two (4%) studied exercise induced laryngeal obstruction (EILO) and two (4%) studied ILO and EILO, paediatric ILO, 20 studies (41%), of which eight (16%) studied ILO, 12 (25%) studied EILO and none studied ILO and EILO and paediatric and adult ILO, 11 studies (22%) of which five (10%) studied ILO, six (12%) studied EILO and none studied ILO and EILO. The median (IQR) sample size was 39 (18–84) participants.

#### 3.2.3 Type of Outcomes

**FIGURE 2:**
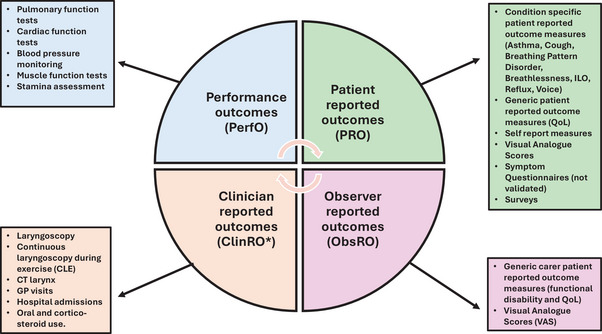
Clinical Outcome Assessments (COAs) used in scoping review.

The outcome assessments were grouped into the four categories of PerfO, ClinRO, PRO and ObsRO measures. A summary of the type of outcome measures used is presented in Table 4. Forty‐eight studies (97%) measured PROs, 15 studies (31%) measured ClinROs, 13 studies (26%) measured PerfOs, and two (4%) measured ObsRO. A visual representation of the type of COAs used is shown in Figure 2.

**TABLE 4:** Clinical outcome assessment (COA) measures used and considerations in line with the WHO‐ICF framework.

	Clinical outcome assessment (COA) used				Outcome measure considerations				
Author	PerfO	ClinRO	PRO	ObsRO	Impairment of body function	Impairment of body structure	Activity limitations and participation restriction	Environmental factors	Personal factors
Baxter et al. 2019	●	●	●		●	●	●	●	●
Baxter et al. 2014		●	●		●	●	●	●	
Campisi et al. 2019	●	●			●		●		
Chiang et al. 2013		●	●		●	●	●		●
Christensen and Thomsen 2011		●	●		●	**●**	●		
De Guzman et al. 2014			●		●	●	●	●	●
DeSilva et al. 2019			●		●	●	●	●	●
Doshi and Weinberger 2006			●		●		●		
Drake et al. 2017			●		●		●		●
Fujiki et al. 2023			●		●		●		●
Fujiki et al. 2024			●		●		●	●	●
Gallena et al. 2015	●		●		●	●	●		
Gallena et al. 2019	●		●		●	●	●	●	●
Gartner‐Schmidt et al. 2014			●		●	●	●	●	●
Gaylord et al. 2020	●		●		●	●	●	●	●
Halevi‐Katz et al. 2019	●		●		●	●	●	●	●
Hatzellis et al. 2012		●	●		●	●	●		
Irewall et al. 2023		●	●		●	●			
Ivancic et al. 2021			●		●	●	●	●	●
Kramer et al. 2017	●		●		●	●			
Kumeresan et al., 2023		●	●		●	●	●	●	●
LeBlanc et al. 2021			●		●	●	●	●	●
Lunga et al. 2022		●	●		●			●	
Maat et al. 2011	●	●	●		●	●	●		●
Marcinow et al. 2014			●		●	●			
Marcinow et al. 2015			●		●				
Mathers‐Schmidt et al. 2005	●		●		●	●			
Maturo et al. 2011			●				●		●
Murry et al. 2006	●		●		●				
Nacci et al. 2011			●		●		●		
Norlander et al. 2015			●		●		●		
Novakovic et al. 2021			●		●	●			
Oda et al. 2022	●	●	●		●	●			
Olin et al. 2022			●		●	●	●		●
Rameau et al. 2012			●		●		●	●	●
Richards‐Mauze and Banez 2014			●	●	●	●	●	●	●
Sandnes et al. 2022			●		●	●	●		
Sandnes et al. 2019a	●	●	●		●	●	●		
Sandnes et al. 2019b	●	●	●		●	●	●		
Schonmman et al. 2023			●		●	●	●		
Shembel et al. 2019		●	●		●	●	●		●
Strojanovic et al. 2022		●	●		●	●	●	●	●
Sullivan et al. 2001			●		●	●	●	●	●
Traister et al. 2016			●		●	●	●		
Vance et al. 2021			●		●	●	●	●	●
Vertigan et al. 2014			●		●	●	●		
Warnes and Allen 2005			●	●	●	●	●	●	●
Yibrehu et al. 2020			●		●		●		
Zalvan et al. 2019			●		●	●	●	●	●

The PROs consisted of validated patient reported outcome measures (PROMs), questionnaires developed for the specific studies, surveys, rating scales and interviews. The validated PROMs used in the studies are shown in Table 5. They comprised of the Asthma Control Questionnaire (ACQ) (Juniper et al. 1999), Asthma Control Test (ACT) (Nathan et al. 2004), Cough Severity Index (CSI) (Shembel et al. 2013), Dyspnoea Index (normal and adapted version) (Gartner‐Schmidt et al. 2014), Laryngeal Hypersensitivity Questionnaire (LHQ) (Vertigan et al. 2014), Nijmegan Questionnaire (Li Ogilvie et al. 2019), Paediatric Quality of Life Inventory (PedsQL) (Varni et al. 2001), Reflux Symptom Index (RSI) (Belfasky et al. 2002), Vocal Cord Dysfunction Questionnaire (VCD‐Q) (Fowler et al. 2015) and Voice Handicap Index (VHI‐10) (Jacobsen et al. 1997). Fourteen studies (29%) used self‐report measures post treatment where patients rated if there had been a change to their symptoms. In Marcinow et al.’s studies (2014 and 2015) patients rated if they had ‘no improvement’, ‘partial improvement’ or ‘complete resolution’ and in Lunga et al.’s study (2022) patients rated if treatment had been ‘successful’ or ‘unsuccessful’. Furthermore, five studies (10%) used visual analogue scores (VAS) where patients rated on a scale the severity of their symptoms pre and post treatment. Moreover, eight studies (16%) developed specific symptom questionnaires or surveys which were collected in a written format or interview pre and post treatment.

**TABLE 5:** Patient reported outcome measures (PROMs) used in scoping review studies.

Questionnaire	Target area	Number of studies measured in	Summary
Asthma Control Questionnaire (ACQ) (Juniper et al. 1999)	Asthma	3	Various versions ACQ‐5, ACQ‐6 and ACQ‐7 5–7 item questionnaire 7‐point Likert scale (0–6) Mean score of all responses calculated. Minimal clinically important change: 0.5
Asthma Control Test (ACT) (Nathan et al. 2004)	Asthma	2	5‐item questionnaire assessing asthma symptoms (daytime and nocturnal), use of rescue medications and the effect of asthma on daily functioning. 5‐point Likert scale (1–5) No minimal clinical important difference established.
Cough Severity Index (CSI) (Shembel et al. 2013)	Cough	2	10‐item questionnaire to assess severity and impact of cough. 5‐point Likert Scale (0–4) No minimal clinical important difference established.
Dyspnea Index (DI) (Gartner‐Schmidt et al. 2014)	Multidimensional breathlessness	11	10‐item questionnaire covering both physical and psychological dimensions. 5‐point Likert scale (0–4) The categories are summed to create the focal score (0–12) Minimal clinical important change: 4
Laryngeal Hypersensitivity Questionnaire (LHQ) (Vertigan et al. 2014)	Laryngeal hypersensitivity	3	14‐item questionnaire covering three domains: obstruction, pain/thermal and irritation, 7‐point Likert scale (1–7) Minimal clinical important change 1.7
Nijmegan questionnaire (Li Ogilvie et al. 2019)	Hyperventilation	1	16‐item questionnaire gives a broad view of symptoms associated with breathing pattern disorder. It was initially developed as a screening tool for hyperventilation. 5‐point Likert scale (0–4). A score over 23 suggests a positive diagnosis of hyperventilation. No minimal clinical important difference established.
Paediatric Quality of Life Inventory (PedsQL) (Varni et al. 2001)	Paediatric QoL	1	23‐item questionnaire to evaluate health‐related quality of life in children 5‐point Likert scale (0–4) No minimal clinical important difference established.
Reflux Symptom Index (RSI) (Belfasky et al. 2002)	Reflux	3	9‐item questionnaire to test laryngo‐pharyngeal reflux disease. 6‐point Likert scale (0–5) A score more than 13 is considered to indicate laryngopharyngeal reflux disease. No minimal clinical important difference established.
Vocal Cord Dysfunction Questionnaire (VCD‐Q) (Fowler et al. 2015)	ILO	2	12 item questionnaire for symptom monitoring in ILO 5‐point Likert scale Score range 12–60 Minimal clinical important difference: 4
Voice Handicap Index (VHI‐10) (Jacobsen et al. 1997)	Voice	2	10‐item questionnaire for assessment of voice 5‐point Likert scale (0–4) A score greater than 11 is considered abnormal Minimal clinical important change: 4

The ClinROs consisted of interpretation of laryngoscopy and continuous laryngoscopy during exercise (CLE), interpretation of CT larynx, measuring GP visits, hospital admissions and oral and inhaled corticosteroid use pre and post intervention.

The PerfOs consisted of pulmonary and cardiac function tests [spirometry, end‐tidal carbon dioxide (ETCO^2^), maximum inspiratory pressure (MIP), forced oscillation technique (FOT) respiratory impedance, Respiratory Tidal Minute Volume (RTMV), heart rate (HR), minute ventilation (VE), maximum rate of oxygen (peak VO_2_), maximal heart rate (MHR), maximal voluntary ventilation (MVV), respiratory rate (RR)], blood pressure (BP) monitoring, airflow perturbation device to measure respiratory resistance and leg fatigue parameters. Sandnes et al. (2022) and Gaylord et al. (2020) also measured the duration of running and distance completed on the treadmill as an objective assessment pre and post intervention and Campisi et al. (2019) measured physical activity levels pre and post intervention.

The ObsROs consisted of validated questionnaires completed by family and/or carers. The functional disability inventory (FDI) (Walker and Greene 1991) and the PedsQL (Varni et al. 2001), carer version.

The time horizon of outcomes ranged from a single one‐off visit measured at two time points (e.g., before and after an intervention), to three years post diagnosis.

#### 3.2.4 Linking Concepts with the ICF Categories

The mapping of meaningful concepts to the ICF categories is shown in Table 4. Some of the outcome measures covered several of the categories. In total, 48 outcomes measured body function, 36 body structures, 40 activity limitations or participation restrictions, 20 environmental factors and 25 personal factors.

#### 3.2.5 Secondary Outcomes

The only phenotypes discussed in these studies were ILO and EILO. There did not appear to be a specific pattern to the outcome measure used depending on patient group (adults or children) or on whether ILO or EILO was measured. Within the EILO cohort (adult and paediatric), the studies used a mixture of PerfO [paediatric EILO, six studies (46%), adult EILO, two studies (15%)], ClinRO [paediatric EILO, six studies (40%), adult EILO, one study (7%)] and PROs [paediatric EILO, 10 studies (21%) and adult EILO, 10 studies (21%)]. Within the ILO cohort (adults and children), the studies used a mixture of PerfO [paediatric ILO, one study (8%), adult ILO, four studies (31%)], ClinRO [paediatric ILO, 0 studies, adult ILO, eight studies (53%)] and PROs [paediatric ILO, 12 studies (26%) and adult ILO, 15 studies (32%)]. More ClinROs were used in the adult EILO and ILO cohort and more PerfOs were used in the paediatric EILO and ILO cohort. The studies that reported ObsROs were both measuring paediatric ILO.

## 4 Discussion

This is the first review to collate methods of ILO measurement from the literature, providing evidence of rapidly increased interest in the field over the last decade and identifying the most used ILO outcome measures. There were 49 papers, discussing 49 studies, containing 27 outcome measures (14 PROs, six ClinROs, five PerfOs, two ObsROs). We have clearly demonstrated the wide variation in outcome measures and the lack of a disease‐specific PRO measure that captures all domains of the WHO‐ICF framework and emphasises the need for outcome set development in this field.

The most used outcome measures were PROs with only one study (Campisi et al. 2019) not measuring a PRO. PROs consisted of validated outcome measures, surveys, rating scales and questionnaires. Most validated PROMs within the studies were generic PROMs and often focused on an alternative disease (e.g., asthma being measured with the ACT and ACQ). Only two studies (Baxter et al. 2019; Strojanovic et al. 2022) used a disease specific PROM, the VCD‐Q. The VCD‐Q focuses on body structure, body function and activity limitations and participation but fails to fully measure contextual factors (environmental and personal). The exercise induced laryngeal obstruction dyspnea index (EILODI) (Olin et al. 2022) is not used as an outcome measure in any the studies of this review but has been validated for use in the EILO cohort (adults and children) and covers all domains of the WHO‐ICF framework so should be considered in future EILO studies.

The studies investigating EILO often used PerfO measures with the most reported outcomes being lung function, heart rate, respiratory rate and blood pressure. Some studies measured exercise capacity and duration alongside the other objective measures. The studies investigating EILO typically used multiple outcome measures, usually testing PerfO, ClinRO and PRO measures in each study.

ClinRO measures consisted of laryngoscopy interpretation pre and post treatment, subjectively assessed by the health care professional completing the test. None of the studies documented if the same health care professional completed the test on the consecutive visits. Only two studies (Baxter et al. 2019, Baxter et al. 2014) reported CT imaging pre and post treatment to monitor laryngeal changes. Some studies discussed medication use pre and post treatment and number of hospital admissions. All studies which collected these data showed a correlation between improved ILO symptoms and reduced hospital attendances/ medication use.

ObsRO measures were collected as proxy reporting in three papers. In the Richards‐Mauze and Banez (2014) paper the FDI parent report was collected alongside the child report. Both child and parent report showed a decrease in functional disability score post intervention. In the Warnes and Allen (2005) paper a VAS rating score on adaptive functioning was given by the mother and child pre and post intervention. Mothers scored a greater reduction in interference of daily functioning by ILO symptoms than the child. Fujiki et al. (2023) used the PedsQL to ask parents about physical, emotional, social and school functioning of their child (Fujiki et al. 2023). The PedsQL score was lower in this population than would be expected at baseline. Therapy was not followed by any specific change in PedsQL score, even in patients who experienced a decrease in dyspnoea. Lower baseline scores predicted more frequent breathing problems six months post therapy.

Several studies, 16/49 (33%) in this review included all outcome measure considerations in line with the WHO‐ICF framework. There were gaps in several of the studies which should be addressed and considered in future research. Most studies reported outcomes that measured body structure and function. This includes documenting structure and function of the vocal cords pre and post challenge, pulmonary function testing, blood pressure monitoring, measuring respiratory resistance and leg fatigue measures. Physical functioning was also measured through questionnaires and surveys such as in the dyspnoea index (DI) which ask patients to rate ‘I feel tightness in my throat when I am having my breathing problem’ (Gartner‐Schmidt et al. 2014) or in the laryngeal hypersensitivity questionnaire asking patients to rate ‘my throat feels tight’ (Vertigan et al. 2014).

Limitations in activity were measured by documenting physical activity levels pre and post treatment and measuring running distances pre and post treatment. Validated questionnaires ask about exercise ability, for example the DI asks patients to rate ‘My shortness of breath gets worse with exercise or physical activity’ (Gartner‐Schmidt et al. 2014), and the ACQ ‘in general, during the last week, how limited were you in your activities because of your asthma?’ (Juniper et al. 1999). Most studies reported that activity levels improved post treatment and a positive association between improved ILO symptoms and activity levels. Richards‐Mauze and Banez (2014) asked parents and children to complete the FDI which includes questions such as rating how difficult it is ‘walking up stairs’, or ‘running the length of a football field’ (Richards‐Mauze and Banez 2014). When using the FDI questionnaire they showed that children and parents both reported a decreased functional disability score post intervention. DeGuzman however demonstrated disagreement between patients and caregivers regarding global improvement on the DI. Patients globally perceived a change post treatment, but carers reported no change post treatment and indicated a slightly lower quality of life for the child (DeGuzman et al. 2014). Participation was reported in several of the validated questionnaire. For example, patients rated on the VCD‐Q how ‘the attacks impact on my social life’ (Fowler et al. 2015); the DI, ‘my breathing problem caused me to restrict my personal and social life’ (Gartner‐Schmidt et al. 2014); and the voice handicap index (VHI‐10) ‘I tend to avoid groups of people because of my voice’(Jacobsen et al. 1997).

Contextual factors (environmental and personal) represent the entire background of a person's life and living situation. Environmental factors make up the physical, social and attitudinal environment in which people live. Personal factors are the background of a person's life and living situation and comprise features that are not part of the primary health condition. Contextual factors can have a positive or a negative influence on a person's function. Environmental factors were not commonly explored or discussed in the included studies. The DI asks patients to rate whether ‘changes in weather affect my breathing problem’ (Gartner‐Schmidt et al. 2014). Visits to GP practices and hospital admissions were captured in a patient survey pre and post treatment. In both studies hospital attendances (taking patients away from their usual environment) reduced significantly post treatment (Baxter et al. 2014; Baxter et al. 2019).

Personal factors which may play a role in treatment outcomes were not explored thoroughly in the included studies. In the VCD‐Q patients were asked to rate ‘I am frustrated! My symptoms have not been understood correctly’ (Fowler et al. 2015), and in the DI patients were asked to rate, ‘my voice problem causes me to lose income’ (Gartner‐Schmidt et al. 2014). Personal factors such as coping styles, self‐esteem, social stigma and change in personality are not explored in detail in any of the studies.

Using the WHO‐ICF framework we have identified that body structure and function and restrictions in activity and participation have been measured extensively but personal and environmental factors were measured in less than 50% of the studies. Measurement of these contextual factors is essential as they have a major role to play in the individuals’ health and return to function.

A recent systematic review by Sandage et al. (2024) looked at intervention strategies used to treat upper airway disorders and the clinical metrics used to determine improvement or resolution of ILO/ EILO. In this review, findings were consistent with this scoping review in the use of clinical metrics and standardised patient perceived outcome measures is highly variable.

In the 49 studies included in this review, there was no consistency in COAs and each study used different outcome measures. There are some ILO‐specific and generic instruments which are used in the studies but the link with the ICF framework was not always considered. This makes comparison across studies difficult and there are currently no minimum standards for ILO research. Some studies had up to 10 primary and secondary outcome measures whereas others had one subjective measure. This review has helped identify gaps in the ILO COAs, demonstrating which ICF categories to focus on future, the need for outcome measures that are ILO specific and standardised ILO outcome sets to compare research outcomes across studies.

### 4.1 Limitations

The scoping nature of the review precluded detailed analyses of reasons behind outcome choices and risk of bias in each study. We did not examine the individual components of each of the specific PROs and whether they met all components of the WHO‐ICF framework, but instead discussed the studies as a whole. Not all surveys were available for review, for example, where a study had developed their own questionnaires. This often made it difficult to determine whether the outcome measure met the domains of the WHO‐ICF framework. Several studies produced their own questionnaires and surveys, suggesting that the authors felt the available validated PRO measures were inadequate. The standardisation of ILO terminology only occurred in 2017 (Halvorsen et al. 2017) but the use of two independent reviewers and software reduced the risk of bias during the screening process. The databases and search terms were chosen after discussion between several reviewers and were felt to cover all terminology prior to 2017 that captured ILO.

## 5 Conclusion

Outcome measures most frequently used for ILO are patient reported questionnaires and surveys, patient performance measures and clinician reported objective assessment. Currently the PRO measures available do not cover all domains of the WHO‐ICF framework and disease‐specific PROs fail to measure personal and environmental impact. Future work should focus on developing core outcome sets for ILO to reduce heterogeneity. Using a common framework for measuring and reporting outcomes ensures consistency and comparability across studies, leading to more reliable and meaningful date in research on ILO. Consensus work involving patients, carers and professionals should be undertaken to ensure future measures are useful and important to the relevant stakeholders.

## Ethics Statement

This study has received ethical clearance from the HRA and Health and Care Research Wales (HRCW). REC reference: 23/NW/0198.

## Conflicts of Interest

The authors declare no conflicts of interest.

## Data Availability

No new data will be created or analysed in this study. Data sharing is not applicable to this article.
